# An Efficient Convolutional Denoising Autoencoder-Based BDS NLOS Detection Method in Urban Forest Environments

**DOI:** 10.3390/s24061959

**Published:** 2024-03-19

**Authors:** Yahang Qin, Zhenni Li, Shengli Xie, Haoli Zhao, Qianming Wang

**Affiliations:** 1 School of Automation, Guangdong University of Technology, Guangzhou 510006, China; yahang.qin@gmail.com (Y.Q.); zhaohli1989@hotmail.com (H.Z.); 2 Guangdong-HongKong-Macao Joint Laboratory for Smart Discrete Manufacturing (GDUT), Guangzhou 510006, China; 3 111 Center for Intelligent Batch Manufacturing Based on IoT Technology (GDUT), Guangzhou 510006, China; shlxie@gdut.edu.cn; 4 The Key Laboratory of Intelligent Information Processing and System Integration of IoT (GDUT), Ministry of Education, Guangzhou 510006, China; 5 The Guangdong Key Laboratory of IoT Information Technology (GDUT), Guangzhou 510006, China; 6 Taidou Microelectronics Technology Co., Ltd., Guangzhou 510006, China; 1112304006@mail2.gdut.edu.cn

**Keywords:** denoising autoencoder, BDS, NLOS, time series features, urban forest

## Abstract

The BeiDou Navigation Satellite System (BDS) provides real-time absolute location services to users around the world and plays a key role in the rapidly evolving field of autonomous driving. In complex urban environments, the positioning accuracy of BDS often suffers from large deviations due to non-line-of-sight (NLOS) signals. Deep learning (DL) methods have shown strong capabilities in detecting complex and variable NLOS signals. However, these methods still suffer from the following limitations. On the one hand, supervised learning methods require labeled samples for learning, which inevitably encounters the bottleneck of difficulty in constructing databases with a large number of labels. On the other hand, the collected data tend to have varying degrees of noise, leading to low accuracy and poor generalization performance of the detection model, especially when the environment around the receiver changes. In this article, we propose a novel deep neural architecture named convolutional denoising autoencoder network (CDAENet) to detect NLOS in urban forest environments. Specifically, we first design a denoising autoencoder based on unsupervised DL to reduce the long time series signal dimension and extract the deep features of the data. Meanwhile, denoising autoencoders improve the model’s robustness in identifying noisy data by introducing a certain amount of noise into the input data. Then, an MLP algorithm is used to identify the non-linearity of the BDS signal. Finally, the performance of the proposed CDAENet model is validated on a real urban forest dataset. The experimental results show that the satellite detection accuracy of our proposed algorithm is more than 95%, which is about an 8% improvement over existing machine-learning-based methods and about 3% improvement over deep-learning-based approaches.

## 1. Introduction

The BeiDou Navigation Satellite System (BDS) is widely used in navigation, geography, and geographic information systems (GISs). For example, it is used to measure the elevation of mountain peaks [1] and evaluate water level changes at different tidal phases [2]. In an open environment, BDS can achieve centimeter-level positioning accuracy. Unfortunately, satellite signals are subject to multipath and non-line-of-sight (NLOS) interference, resulting in large positioning errors that often occur suddenly in complex urban environments [3,4]. BDS signals are emitted from satellites, propagate through space and the atmosphere, and finally reach the receiver. During this process, multiple sources of error lead to inaccurate positioning. These errors can be categorized into three main types related to satellites, signal propagation paths, and receiving equipment (i.e., transmitters, propagators, and receivers), for example, the error [5,6,7] generated when the signal is penetrating through the ionosphere and troposphere, the error caused by the Doppler effect of high-speed satellite movement, the error of the multipath effect, the error of the satellite clock, the error of ephemeris (satellite position and speed), the error of internal noise, etc. Most of the errors can be eliminated or weakened by using the short-baseline double difference (SBDD) technical [8]. SBDD positioning methods require the receiver to be very close to one or more reference stations, usually no more than a few tens of meters. Because the baseline is short, the effect of errors is small, and high-accuracy positioning can be achieved under relatively simple conditions. Meanwhile, satellite clock and receiver clock errors are eliminated, and ephemeris errors, ionospheric delays, tropospheric delays, and other spatially correlated errors are greatly weakened. However, NLOS cannot be eliminated and has become a major problem for inaccurate BDS positioning.

**Figure 1:**
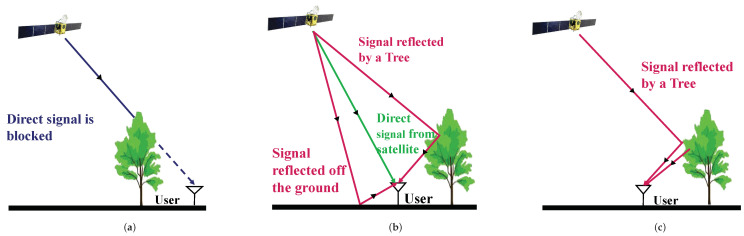
Three satellite signal types of LOS, multipath, and NLOS in an urban forest environment. (**a**) Blocked satellite; (**b**) multipath satellite; (**c**) NLOS satellite.

Figure 1 shows satellite signal categories in an urban forest environment [9]. Specifically, the satellite signal in Figure 1a is completely obscured by trees and cannot be received by the receiver. In Figure 1b, a multipath signal occurs when the satellite signal is reflected by nearby trees, causing interference with the direct signal. Figure 1c shows the NLOS signal, where the receiver can only pick up signals reflected or scattered by nearby trees, which can produce positioning errors of hundreds of meters [10,11]. Therefore, multipath interference in urban forest environments has become an important research topic in BDS urban positioning.

Recently, artificial intelligence (AI) methods have gained a lot of attention, and most of them focus on supervised learning methods to recognize multipath and NLOS, e.g., k-nearest neighbor (KNN) [12], support vector machines (SVM) [13], decision tree (DT) [14], and convolutional neural network (CNN) [15] methods. These supervised learning methods are able to automatically acquire potential high-dimensional features for multipath and NLOS, achieving high recognition accuracy. However, it requires the construction of datasets containing a large number of labels, which is expensive and time-consuming. To alleviate the above problems, a multipath identification method based on unsupervised K-means [16,17,18] is proposed. However, this unsupervised machine learning (ML) method is less effective than supervised learning methods in terms of recognition accuracy.

**Figure 2:**
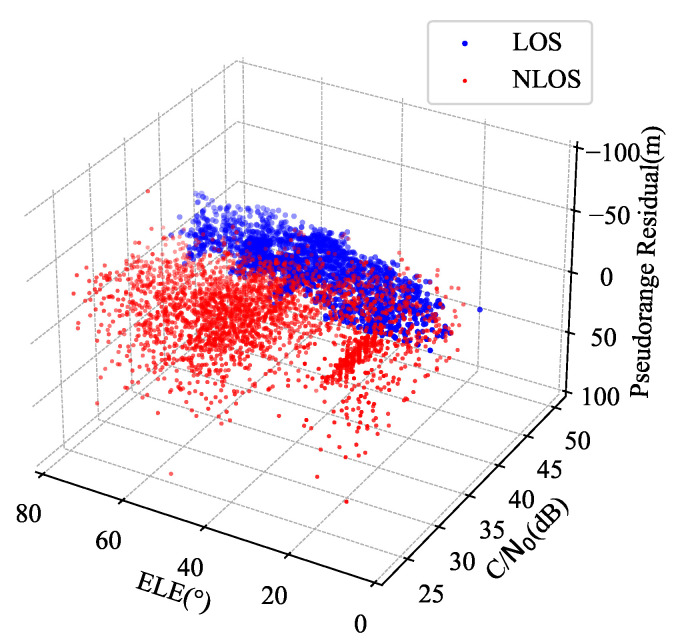
Point cloud of RINEX-level features of BDS signals.

In harsh urban forest environments, which are affected by dense foliage, the BDS signal propagation direction is complex and variable, making the extracted features linearly indivisible [19]. We used an u-blox F9 receiver to receive the data in RINEX format; the exact way that the data were collected is described in detail in Section 5. Figure 2 shows a 3D point cloud of the carrier-to-noise ratio ($C/N_{0}$), the elevation angle (EA), and the pseudorange residual (PR) of the signal, where blue represents the LOS signal and red represents the NLOS signal. Ideally, the two signals should be completely separate, i.e., they should have distinct boundaries. However, they are now partially mixed together due to noise interference. We aim to distinguish between different signal types using our proposed method.

In addition, current learning-based methods suffer from difficulties in constructing a large number of label databases, low model detection accuracy, and poor generalization. Furthermore, the above works discuss urban canyon environments only, so  NLOS detection in urban forest environments is still an open problem.

From a technical standpoint, we mainly address the following challenges:

(1) How to find representative features automatically with the help of unlabelled data. Current NLOS signal detection methods based on supervised learning have the problem of the difficulty of constructing a large number of labeled databases.
(2) How to improve the generalization and robustness performance of NLOS detection models. Current unsupervised-learning-based NLOS signal-detection methods usually use a model of clustering, which cannot fully understand the complex relationship between the data, resulting in a low model detection accuracy. Furthermore, the different environmental noises around the receiver lead to a robustness that needs to be improved.

**Figure 3:**
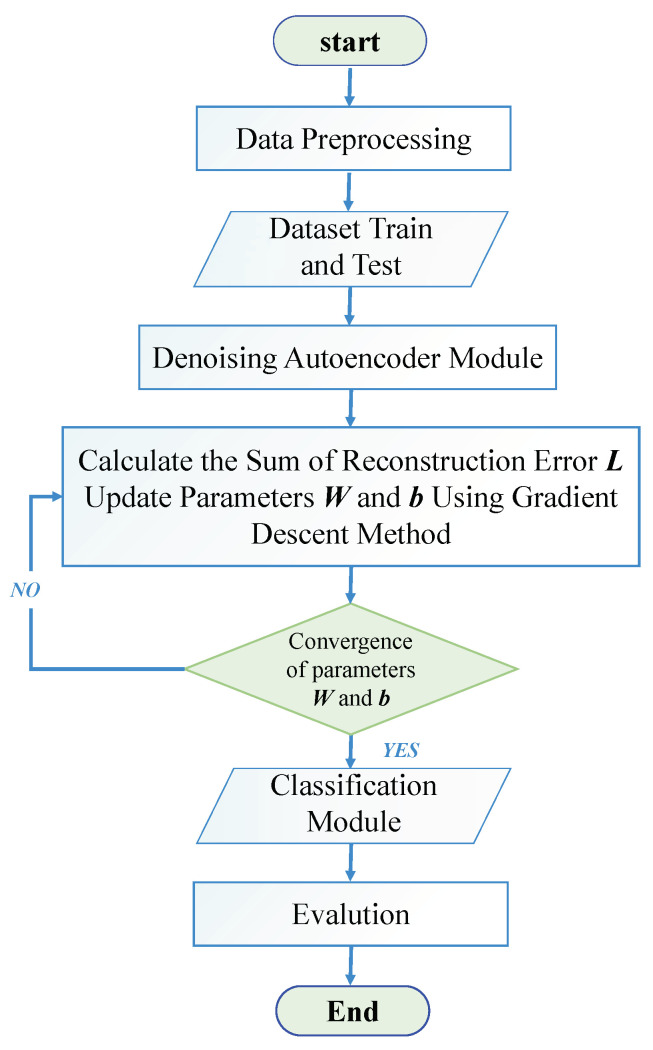
The flow chart of the proposed CDAENet training algorithms.

To overcome the above difficulties, in this article, inspired by the success of deep learning (DL) techniques, we proposed a novel NLOS multipath detection method, i.e., the convolutional denoising autoencoder (CDAENet). Unsupervised feature learning can effectively solve the problem of a small number of labels. It trains the model by using a small number of labeled samples and a large number of unlabeled samples at the same time so that the model has sufficient learning ability in the case of a small number of labels. At the same time, we add noise to the data to reduce the model’s dependence on inaccurate input data, which in turn increases the model’s generalization performance and robustness. In detail, the flow chart of the CDAENet training algorithms is shown in Figure 3. First, we introduce deep DAE to capture the low-dimensional features of the BDS data, achieving dimensionality reduction while providing robust signal reconstruction. Speed is important for BDS real-time localization. As our input data are high-dimensional time series data, the computation of the model can be effectively reduced with dimensionality reduction. The reliability of the recognition results and the robustness of the model are improved by incorporating Gaussian noise. As far as we know, this is the first study using DAE for BDS recognition applications. Then, to further improve the detection accuracy of the model, we propose a multi-layer perceptron (MLP) model to capture the nonlinear correlation of NLOS. Finally, we verify the superiority of the proposed model in a real urban forest environment. The experimental results show that our method obtains more accurate detection results than previous methods.

The main contributions of the proposed CDAENet for BDS NLOS signals detection are listed as follows:

1. To improve the NLOS detection performance in urban forest environments, we develop an efficient CDAENet method that introduces $C/N_{0}$, PR, and EA as input features to achieve the detection of NLOS signals with long time series.
2. To solve the difficulty of constructing a large number of labeled databases, we use the DAE module to learn low-dimensional representation in time series data, achieve efficient unsupervised feature extraction, and provide robust signal reconstruction. Meanwhile, the robustness of the model is improved by adding noise.
3. To further improve the detection accuracy of the model accurately and effectively, we use the MLP module to identify compressed features and utilize the nonlinear modeling ability of MLP to increase the generalization performance of the model.

The remainder of the article is organized as follows. Section 2 reviews the related work. Section 3 introduces the features of BDS data in depth. Section 4 elaborates on the specific structure of our proposed CDAENet and introduces component modules in the context of a general fusion network. Section 5 describes the data-collection method. In Section 6, we discuss the extensive experiments and analyses under different environments. Finally, we conclude the article and provide plausible future research work in Section 7.

## 2. Related Work

Due to the complex urban forest environment and uncertain noise background, separating the two satellite signals, LOS and NLOS, is still a challenge. In recent years, various algorithms have been proposed to resolve this dilemma, which can mainly be classified into three categories: (1) multi-sensor fusion methods, (2) machine-learning-based methods, and (3) deep-learning-based methods.

Multi-sensor fusion methods. Methods in the first category use a comprehensive solution including the global navigation satellite system (GNSS) and multi-sensor integration. Several methods have been extensively explored for positioning in multipath and NLOS environments using sensors such as light laser detection and ranging (LiDAR), sky-pointing cameras, and inertial measurement units (IMU) in combination with GNSS. In addition, recent studies [20,21,22,23] have utilized dynamic users at multiple locations on the road to generate multipath maps and employed residual-based algorithms to address location inaccuracies in urban canyon environments. Nagivation applications integrating a cubature Kalman filter (CKF) method and strap-down inertial navigation system (SINS) have been proposed to improve the navigation error under harsh GNSS environments [24]. However, this approach usually requires additional hardware devices, which not only increase the size of the BDS application but also increase the cost. As a result, the applicability of BDS positioning is severely limited and is not suitable for low-cost navigation services. The sensors could work well under different environmental conditions, such as volatile organic compounds monitoring and indoor gas monitoring [25]. Meanwhile, in outdoor environments, some sensors are more sensitive to light and weather, making them unsuitable for positioning in many complex scenarios. Among them, [26] found that raindrops have negative effects on LiDAR performance. In very clean sky conditions, e.g., after rain brings aerosols to the ground, the returning signal will be too weak for LiDAR to provide meaningful estimates when aerosol particles are absent. Ref. [27] pointed out that short-duration oscillations can be clearly observed by our sensor due to the influence of wind.

Machine-learning-based methods. Methods in the second category consider learning-based approaches to detect multipath and NLOS. GNSS measurement errors in complex environments are difficult to fully model due to the influences of multipath and NLOS. Learning-based methods are trained using only data, avoiding strict assumptions about the structure of the data. Several studies have proposed supervised ML methods. Scholars have proposed the detection of $C/N_{0}$, EA, AA, PR, and pseudo-distance residual consistency (PRC) using SVM [13], extreme gradient boosting (XGBoost) [28], random forest (RF) [29], DT [14], and KNN [12] methods, respectively. The above ML methods have their own advantages and limitations. Ref. [30] proposed a two-stage stacked integrated learning (SEL) method that combines the three ML methods. The first level uses SVM and XGBoost, and the second level uses logistic regression (LR) to detect NLOS. A series of works [16,17,18] used an unsupervised method of K-means clustering to detect multipath/NLOS. However, ML-based methods have poor generalization performance in different environments, resulting in the detection accuracy of current ML methods still needing to be improved.

Deep-learning-based methods. Methods in the third category suggest that the DL can effectively solve nonlinear problems andq43is widely used for the identification of BDS signals. For example, ref. [15] proposed a CNN multipath-signal-mitigation method. Experimental results show that the method achieves relatively good localization results in both dynamic and static environments. In [31], a DL model is proposed for the first time to detect GNSS image multipath signals in the time-frequency domain. Furthermore, CNN is used to identify NLOS signals in the correlation output. This approach adds weights based on NLOS probabilities to PR to improve GNSS positioning in complex urban environments [32]. Ref. [33] developed a deep neural network (DNN) to address the limitations of traditional beamforming methods and improve the positioning performance of vehicle navigation in harsh multipath environments. Meanwhile, a model for NLOS signal detection and correction based on CNN and variational modal decomposition is proposed. The model first uses CNN to identify the NLOS signal. Then, the variational mode decomposition method is used to decompose the detected NLOS signals and eliminate the NLOS part [34]. Based on image characteristics, a CNN model is proposed to estimate images with synthetic multipath information generated by a GNSS signal-propagation simulator [35]. However, DL-based NLOS detection requires a large number of labeled data. In addition, in the process of NLOS detection, there are still some problems, such as high dimensional data redundancy, low detection accuracy, and poor generalization performance.

Although these model-based methods have obtained promising detection results, they are still inadequate in terms of NLOS series information. The specific limitations can be broadly summarised as follows: (1) Supervised-learning-based NLOS detection techniques require a large number of labeled data, yet constructing large-scale labeled data is difficult. (2) The data are contaminated by the noise around the receiver and exhibit non-linear characteristics, and the detection accuracy and robustness of the existing learning-based NLOS models are low. To address the above limitations, from the perspective of series data, we applied DAE to achieve unsupervised NLOS detection. Then, Gaussian noise was added to the model to improve its robustness. Finally, MLP was used to detect nonlinear NLOS data.

## 3. Features Selection and Preprocessing

The raw observations of BDS include *C*/$N_{0}$, PR, EA, carrier phase, and Doppler shift [36]. These raw observation parameters can be used as the features for ML, and we selected three of these representative features as input data.

### 3.1. Features Selection

(1) Carrier-to-Noise Ratio (*C*/$N_{0}$): The *C*/$N_{0}$ is a basic indicator of the quality of radio frequency (RF) signals such as BDS, indicating the strength of the received signal. Signals with stronger *C*/$N_{0}$ possess better positioning performance. However, in urban forest environments, signal strength is reduced due to LOS superimposed on NLOS such as reflection and refraction. It can be calculated using the following equation:

(1) $$C/N_{0}=A_{d}^{2}+A_{r}^{2}+A_{m}^{2}+2A_{d}^{2}A_{r}^{2}A_{m}^{2}\cos\Delta \phi$$

where $A_{d}$, $A_{r}$, and $A_{m}$ are the amplitudes of the LOS, NLOS, and MP signals, respectively; ϕ is the phase; and Δϕ is the phase shift of the NLOS. As shown in Equation (1), the strength of the BDS signal is closely related to the strength of the NLOS and MP signals.

Here, we used $C/N_{0}$ instead of the signal-to-noise ratio (SNR). This is because SNR is the ratio between the power of a signal or carrier and the power of noise in a given specific bandwidth. Hence, we need to have the same bandwidth to have a fair SNR comparison between different signals. $C/N_{0}$ is the ratio of the carrier power to the noise power per unit of bandwidth, independent of receiver bandwidth, and can be used directly to indicate the quality of the received signal.

(2) Pseudorange Residual (PR): The PR represents the geometric distance between the satellite and the ground user reception position, and it is usually calculated using the weighted least squares (WLS) method.

(2) $$\Delta x={\left(H^{T}WH\right)}^{-1}H^{T}W\Delta \rho$$

where Δx denotes the receiver state, which consists of the 3D position difference between the receiver and the satellite and the receiver clock error. *H* is the observation matrix of the satellite, ρ is the pseudo-distance measurement vector, and *W* is a weighting matrix. According to Equation (2), the PR for each satellite can be calculated as follows:

(3) PR=Δρ−HΔx

In the case of static receivers, the PR for LOS is slightly jittery around zero, and the PR for MP and NLOS is usually large.

(3) Elevation Angle (EA): The satellite elevation angle can be used as one of the important metrics for signal identification. EA can be calculated using Equation (4):

(4) $$EA=\sin^{-1}\left({\hat{r}}_{U}/∥\hat{r}∥\right)$$

where $\hat{r}\in R^{3}$ is the estimated distance between the satellite and the receiver in the northeast-up (ENU) coordinate system. ${\hat{r}}_{U}$ is the “Up” component of *r*.

**Figure 4:**
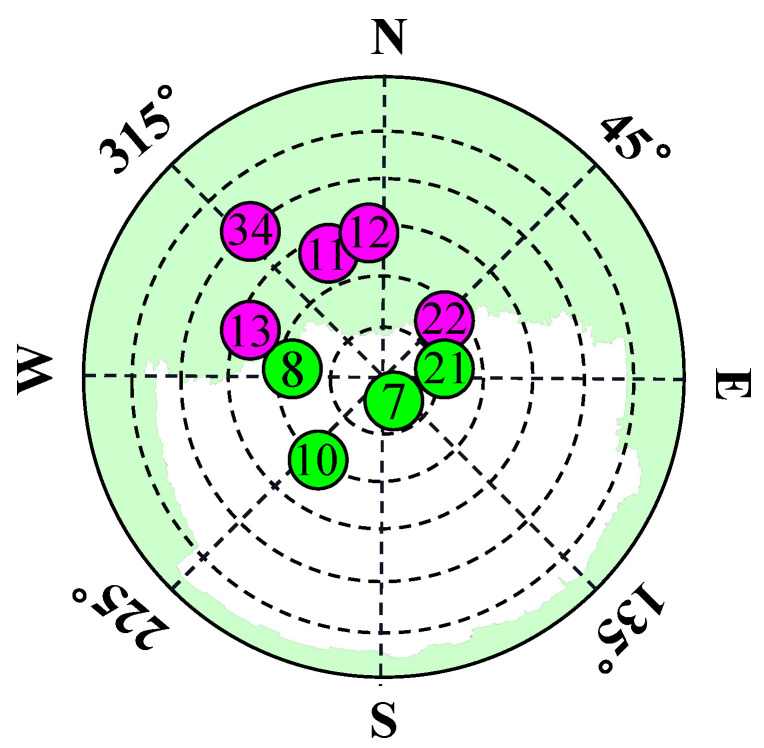
Two types of BDS signal: LOS only includes direct signals and NLOS caused by only diffracted/reflected signals from “invisible” satellites. (Green is the LOS satellite, and pink is the NLOS satellite).

There is a strong correlation between the occlusion of GNSS signals and the corresponding satellite EA. As shown in Figure 4, satellite signals at lower elevations are more likely to be NLOS satellites. On the contrary, satellite signals at higher elevations are more likely to be LOS satellites. In addition, according to the law of reflection, signals at higher elevations are more difficult to reflect because they require the building to be high enough to have an effective reflection point.

### 3.2. Normalization of Features

To improve the convergence speed and detection performance of the proposed model, the maximum-difference normalization method is used to transform the original data samples with dimensions into dimensionless expressions so that their values are concentrated between 0 and 1. The normalization formula can be expressed as follows:

(5) $$x_{i}=\frac{x-x_{min}}{x_{max}-x_{min}}$$

where $x_{i}$ is the normalized input data, *x* is the raw unprocessed data, $x_{min}$ and $x_{max}$ are the minimum and maximum values in the raw data, respectively.

## 4. An Efficient Convolutional Denoising Autoencoder Method for BDS NLOS Detection

Our primary goal is to identify the BDS NLOS in an urban forest environment. Therefore, a model called CDAENet, based on a denoising autoencoder (DAE) mechanism, was established to obtain dimension features. First, we give the overall structure of the proposed CDAENet model. Second, we introduce the architecture of the DAE. Finally, the MLP-based detection algorithm is described.

### 4.1. Overall Network Structure

**Figure 5:**
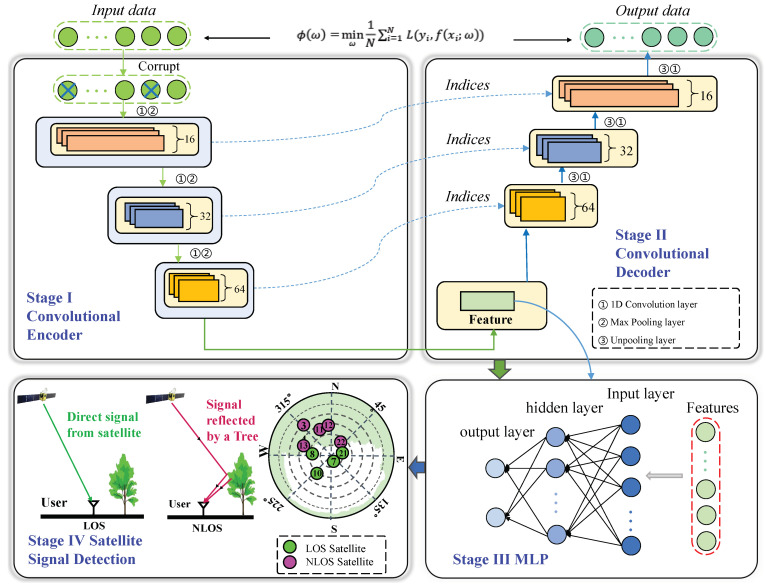
The detailed framework of the proposed CDAENet algorithm for NLOS detection.

We focus on developing a generic DL network for NLOS detection. Figure 5 shows the overall network framework of CDAENet. Intuitively, the proposed CDAENet is jointly trained in an end-to-end manner and consists of two main sub-modules containing the DAE and MLP classifier components. The original data are corrupted by incorporating some noise components to make the model more robust, the encoder achieves feature extraction along with data dimensional compression, and the reconstructed input data are generated by the decoder. The decoder is similar to the encoder part in that the sigmoid is chosen as the activation function for the hidden layer of the model. Meanwhile, we employ a convolutional operation to extract local features of the input signal, thus helping the algorithm to understand the data better. Finally, the MLP model is used to further improve the detection performance.

### 4.2. Proposed Denoising Autoencoder Module

#### 4.2.1. Autoencoder

**Figure 6:**
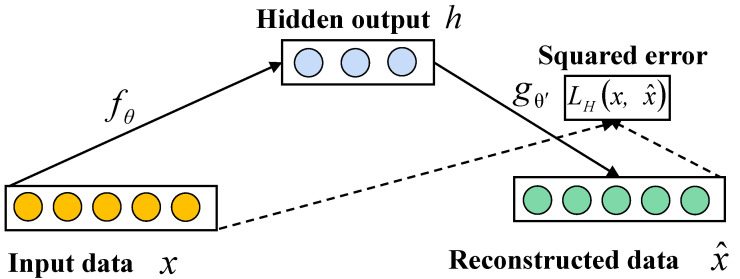
The overall structure of an autoencoder.

Autoencoder is a type of artificial neural network trained in an unsupervised manner, learning to reconstruct its original input. An autoencoder consists of two parts: an encoder and a decoder, as shown in Figure 6. The encoder compresses the input *x* into a low-dimensional potential space representation *h* using a nonlinear transformation. The decoder reconstructs the original input data by means of a potential space representation *h*. This process can be expressed as follows:

(6) $$h=f\left(Wx+b\right)$$

(7) $$\hat{x}=g\left(W^{\prime }h+b^{\prime }\right)$$

where $\hat{x}$ denotes the decoder estimation vector and *f* and *g* denote the nonlinear activation functions of the encoder and decoder, respectively. *W* and $W^{\prime }$ denote the weight matrices of the encoder and decoder, respectively. *b* and $b^{\prime }$ denote the bias vectors of the encoder and decoder, respectively. The overall network is trained using backpropagation to make *x* as close to $\hat{x}$ as possible, thus updating of parameters $\theta =\left\{W,b,W^{\prime },b^{\prime }\right\}$.

Autoencoders have shown superior effectiveness in dimensionality reduction, target detection, image classification, and image denoising. Variational autoencoders (VAEs) have been widely used as enhanced autoencoders in the field of image processing and text generation [37]. An overview of the performance of VAEs in source separation, finance, bio-signal applications, target detection, image classification, and image denoising is presented [38]. Ref. [39] explored the applicability of the VAE model in modern game design.

Inspired by the above autoencoder and variational autoencoder, we developed a denoising autoencoder to recognize NLOS signals in an urban forest environment.

#### 4.2.2. Dignoising Autoencoder

**Figure 7:**
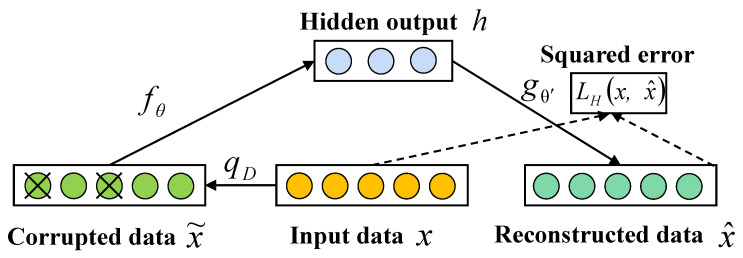
The overall structure of a a denoising autoencoder.

For the autoencoder model, if the input contains a certain amount of noise, the reconstructed data cannot recover the input information completely. In other words, the features of the input data learned by the AE model are susceptible to noise. As a nonlinear feature extraction and dimensionality reduction technique, DAE [40] can eliminate noise and redundant information from high-dimensional spaces, thereby providing a useful low-dimensional input representation. Specifically, DAE is a deformed model that adds noise to the autoencoder. The dependence on the input signal is reduced by modeling the loss of information. The feature learning ability of the hidden layer is enhanced by introducing random noise in the input and forcing the autocoder to remove the random noise during the learning process, thus reducing its sensitivity to the input samples. The DAE improves the robustness of the encoder to complete the learning of input signals containing noise while improving its generalization ability and preventing training overfitting [41], the structure of which is shown in Figure 7. A denoising autocoder is similar to an autocoder and mainly consists of an encoder and a decoder. There is an encoder that creates a compressed representation. This representation goes into the decoder and outputs a reconstructed input [37,38,39]. In addition, the denoising autocoder adds extra noise to the input section to increase the robustness of the model.

The DAE is mainly designed to learn more generalized features from input data containing noise, and its training process is as follows:

Step 1: The process of destroying the initial input *x* by random mapping to obtain the datapoint $\tilde{x}$, which contains noise, can be expressed as follows:

(8) $$\tilde{x}∼q_{D}\left(\tilde{x}|x\right)$$

The purpose of adding noise is to hope that the model learns to withstand some random perturbations and become less sensitive to inputs. Reduced sensitivity usually means that the model is less dependent on the training set, which will help the model perform better on new data. Therefore, it helps to improve the generalization performance of the model. The probability density function of Gaussian noise follows a Gaussian distribution, which can be expressed as:

(9) $$f\left(x\right)=\frac{1}{\sigma \sqrt{2\pi }}exp\left(-\frac{{\left(x-\mu \right)}^{2}}{2\sigma^{2}}\right)$$

where μ is the expectation and σ is the standard deviation.

Gaussian noise is used to better simulate unknown real noise. In a real environment, noise is often caused not by a single source but by a complex of many different sources of noise. If we consider real noise as the sum of many random variables with different probability distributions, each of them is independent. According to the central limit theorem, their normalized sum approaches a Gaussian distribution as the number of noise sources increases. *S* is the observed noise and can be expressed as

(10) $$S=\sum_{i=1}^{n}r_{i}$$

where $r_{i}$ is one of a large number of random noises.

Step 2: The encoder maps the input values $\tilde{x}$ into a hidden representation *h* by means of an activation function. The process is identical to that of a conventional autoencoder, defined as follows:

(11) $$h=f_{\theta }\left(\tilde{x}\right)=s\left(W\tilde{x}+b\right)$$

where *s* is a nonlinear activation function and *W* and *b* are the weights and bias of the hidden layer, respectively.

Step 3: The decoder uses an activation function to decode the hidden representation *h* to the output $\hat{x}$, defined as follows:

(12) $$\hat{x}=g_{\theta^{\prime }}\left(h\right)=s\left(W^{\prime }h+b^{\prime }\right)$$

where *s* remains a nonlinear activation function, $W^{\prime }$ and $b^{\prime }$ are the weights and bias of the output layer, respectively. The parameters of a DAE are $\theta^{\prime }=\left\{W,b,W^{\prime },b^{\prime }\right\}$.

The sigmoid activation function adopted in this article can be expressed as

(13) $$f_{\theta }\left(x\right)=g_{\theta^{\prime }}\left(x\right)=\frac{1}{1+e^{-x}}$$

The network is trained to make the input *x* as close to the output $\hat{x}$ as possible. To obtain the model parameters, the Mean Square Error (MSE) loss function can be obtained by minimizing the mean loss function $L\left(\cdot \right)$ error:

(14) $$L\left(W,W^{\prime },b,b^{\prime }\right)=\frac{1}{2N}\sum_{t=1}^{N}{∥{\hat{x}}_{t}-x_{t}∥}^{2}$$

(15) $$=\frac{1}{2N}\sum_{t=1}^{N}{∥g_{\theta \prime }\left(x_{t}\right)-x_{t}∥}^{2}$$

### 4.3. Proposed Convolutional Denoising Autoencoder Algorithm

We developed the CDAENet method to detect LOS/NLOS categories in real urban forest datasets. First, the DAE can remove the noise while downgrading the dimensions. Second, MLP aim at capturing nonlinear relationships by superimposing multiple neural networks. Therefore, we used multiple fully connected layers to learn the nonlinear trend of the NLOS signal. Finally, test samples are fed into the trained CDAENet classifier to detect NLOS. See Algorithm 1 for details.
**Algorithm 1** CDAENet algorithm for NLOS recognition in the urban forest environment.**Input:** The raw dataset D1, D2, D3, and D4. (D1, D2, D3 and D4 correspond to the four urban forest environments and more details can be found in Section 5.2)**Output:** The type of the BDS signal.$h_{j}^{l}=\left[h_{1},h_{2},\ldots h_{M}\right]\in M^{l}$, where $h_{i}$ is the number of hidden units in layer *i* and *l* is the number of hidden layers.$\hat{x}=\left[{\hat{x}}_{1},{\hat{x}}_{2},\ldots {\hat{x}}_{z}\right]\in Z^{l}$, where ${\hat{x}}_{i}$ is the number of output units in layer *i* and *l* is the number of output layers.1:**for** iterator=1,2,3,…,T **do**2:    Generate latent variables matrix *H* through Equation (11)    $h=f_{\theta }\left(\tilde{x}\right)=s\left(W\tilde{x}+b\right)$3:    Let the loss J be defined as (using Equation (14))    $L\left(W,W^{\prime },b,b^{\prime }\right)⟵\min_{\omega }\frac{1}{2N}\sum_{t=1}^{N}{∥{\hat{x}}_{t}-x_{t}∥}^{2}$4:    Compute stochastic gradient of the loss w.r.t each ω    $▽\bar{L}={▽}_{\theta ,\phi }\left(\frac{1}{\left|M\sum L_{i}\right|}\right)$5:    Update network parameters by backpropagation    $\left(\theta ,\phi \right)⟵$ Stochastic gradient descent optimizer6:**end for**7:**for** $h_{j}^{l}=\left[h_{1},h_{2},\ldots h_{M}\right]\in M^{l}$**do**8:    For detection on the data $h_{j}^{l}$ using MLP9:**end for**10:**return** LOS or NLOS

## 5. BDS Data Description

To investigate the effectiveness of the proposed CDAENet method for NLOS detection, this article collected data in a real urban forest environment. This part describes in detail the data-collection equipment, the data labeling method, and the data format.

### 5.1. Data Collection

**Figure 8:**
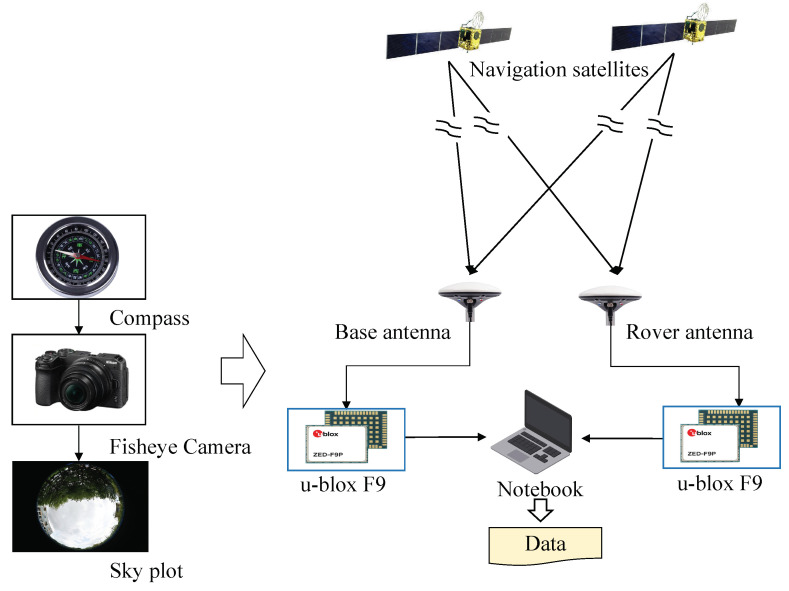
The installment of the hardware equipment for BDS signal collection.

Figure 8 shows the BDS signal collected device, which consists of an antenna, a u-blox F9 GNSS receiver, a laptop, a fisheye camera, and a compass. The collection process consists of two main steps, i.e., (1) the taking of sky pictures by a fisheye camera, and (2) the collection of data from the BeiDou satellite. In detail, we first used a compass to determine the north, adjust the position of the fisheye camera to align with the north, and take an image of the sky view. Then, the antenna, the high-accuracy positioning module, and the laptop computer were connected according to Figure 8, and the computer program was adjusted to collect the raw data of the BeiDou satellite signal in RINEX format. Herein, to collect more accurate positioning data, we used the double differential approach. So we used two antennas, one as a reference station and the other as a mobile station. Finally, the collected .dat file is solved using RTKLIB 2.4.2.

The detailed solution process is as follows. First, the u-center and serial assistant are debugged to obtain the raw .dat format file, which is stored on the computer. Second, the data are decoded using the rtkconv module in RTKLIB to obtain the GNSS observations and store them in the common RINEX format. In detail, the file header format, satellite system, etc. are configured through the Options option. When all options are configured, “Convert” is clicked to start the conversion of the file format to RINEX. Furthermore, the data are decoded using the rtkconv module in RTKLIB to obtain the GNSS observations and stored in the common RINEX format. Specifically, the file header format, satellite system, etc., are configured through the “Options”. When all options are configured, “Convert” is clicked to start the file format conversion to RINEX. In addition, the $C/N_{0}$ can be observed directly from the raw data. The EA and AZ are calculated after calculating the satellite position and station position, and the PR is calculated based on the satellite position, station position, and atmospheric correction. Finally, the satellite position, EA, AZ, and PR can be calculated by running “Options” in the rtkpost module of RTKLIB to select the input file, configure the observation parameters such as positioning mode and frequency type, and click the “Convert” button to start the process of solving the observation data.

### 5.2. Location of Data Collection

**Figure 9:**
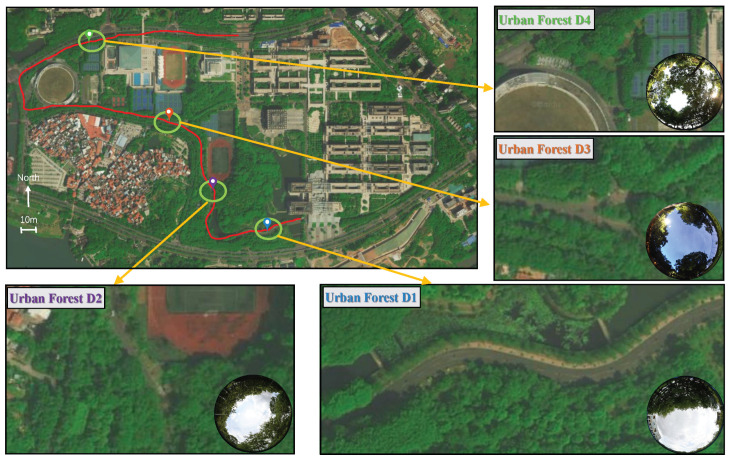
Data-collection environment of D1, D2, D3 and D4.

Data were collected on the boulevard path at Guangdong University of Technology using the equipment in Figure 8, and we give a sky map for each location. In this case, the first location for data collection was at the beginning of the road, along which locations B, C, and D were identified, and the walking trajectory is shown in Figure 9. The collection site contained several typical environments, such as single-sided, double-sided, triple-sided, and quadruple-sided occlusion situations. The streets were densely populated on both sides with tall trees that were more than 10 m, and the data were collected on 20–23 July 2023. At this time, the leaves grow very thick, and there are almost no gaps. In particular, this is a strongly reflective environment for satellite signals. This environment is common in urban areas, and it is prone to NLOS reception. Location A is the unilateral foliage case, location B is the two-sided foliage case, location C is the three-sided foliage case, and location D is the four-sided foliage case. These data-collection environments are shaded only by foliage and not by other buildings (e.g., high-rise buildings and viaducts).

### 5.3. Data Labeling

**Figure 10:**
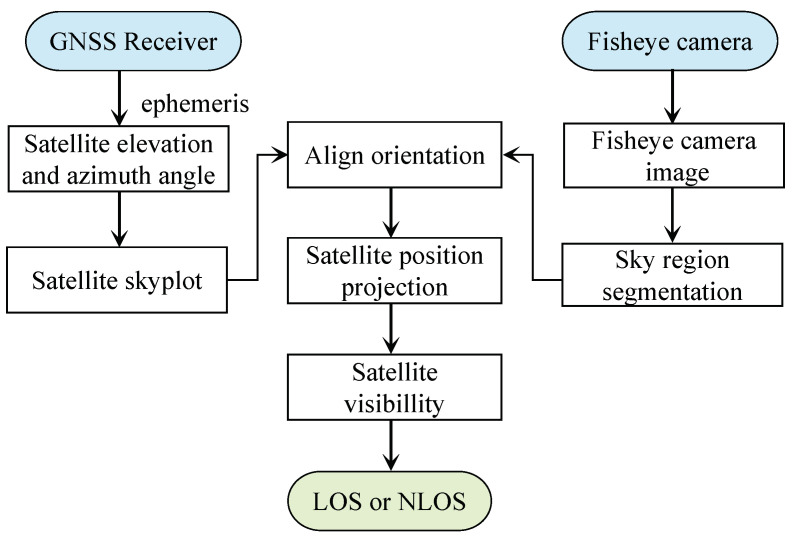
Flow of determining the type of BDS signal using fisheye images.

**Figure 11:**
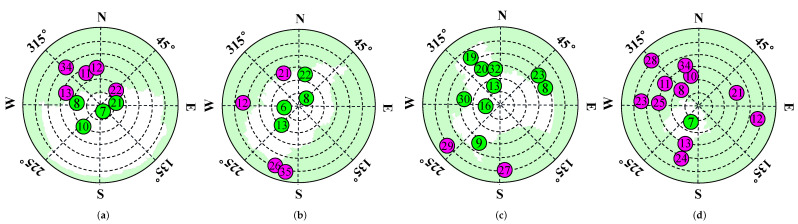
Sky plot of different locations with the satellite visibility labeled from ground truth. (**a**) D1; (**b**) D2; (**c**) D3; (**d**) D4. (Green is the LOS satellite, and pink is the NLOS satellite).

To validate the proposed CDAENet algorithm, it is necessary to collect satellite signal data from a real environment. Inspired by the literature [42], signal types were labeled using a method of projecting satellite positions from images taken by an eye camera. For each satellite measurement provided by the receiver, the elevation and azimuth angles of the corresponding satellite were calculated. The elevation and azimuth angles are projected onto a skyplot of the satellite to determine the satellite signal type. The detailed methodology can be found in Figure 10. The results of the labeling of the data are shown in Figure 11, where the green color in the skyplot represents trees, and the satellites falling in them are NLOS signals. The white color represents the sky area, and the satellites falling in it are LOS signals.

### 5.4. Time Series Data

**Figure 12:**
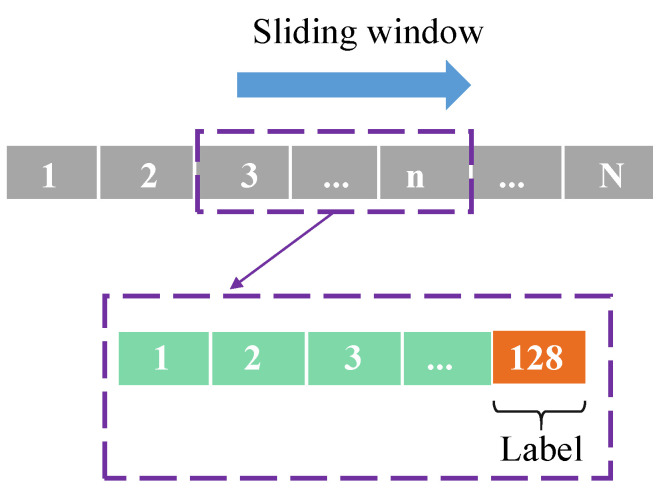
Data sliding window of D1, D2, D3, and D4.

To satisfy the required data dimensionality of the CDAENet, we use a sliding window to segment the data. As shown in Figure 12, the time series length is chosen to be 128 s, and the sliding window step is taken to be 10 s. The window length is chosen in this way mainly because a compact set of tokens is sufficient to represent the changed semantic concepts of interest, while redundant tokens may hamper the performance of the model. When the window is too short, the model may lose some useful information related to the changed concept. In addition, the small step size may lead to a high degree of similarity between the two windows, which will reduce the recognition accuracy of the model.

## 6. Experimental Results

In this section, we analyze in detail the impact of each component on the CDAENet model. Specifically, we present the experimental setup, including parameter settings, evaluation metrics, and comparison models. Furthermore, a multifaceted analysis of the model is performed for comparison, including hyperparameter tuning, model validity comparison, visualization result analysis, and model performance analysis under the influence of noise.

### 6.1. Implementation Details

Our network was performed with the popular DL framework TensorFlow version 2.12.0 and Windows 10 operating system, the programming language used was Python 3.9, and we used the Adam optimizer to train the models. All experiments were implemented on an 11th Gen Intel(R) Core(TM) i5-11500 @ 2.70GHz 2.71 GHz CPU with a batch size set to 200. For each dataset, we selected 70% as the training set and 30% as the test set.

### 6.2. Evaluation Metrics

We used accuracy (Acc), F1-score (F1), precision (Pre), and recall (Rec) [43] to evaluate the performance of the model. All four evaluation metrics are based on a confusion matrix, which includes true positive (TP), false positive (FP), false negative (TN), and false negative (FN). The evaluation metrics can be calculated as follows:

(16) $$\mathrm{Accuracy}=\frac{\mathrm{TP}+\mathrm{TN}}{\mathrm{TP}+\mathrm{TN}+\mathrm{FP}+\mathrm{FN}}$$

(17) $$F1\text{-}\mathrm{score}=2\frac{\mathrm{recall}\times \mathrm{precision}}{\mathrm{recall}+\mathrm{precision}}$$

(18) $$\mathrm{Precision}=\frac{\mathrm{TP}}{\mathrm{TP}+\mathrm{FP}}$$

(19) $$\mathrm{Recall}=\frac{\mathrm{TP}}{\mathrm{TP}+\mathrm{FN}}$$

which are introduced in detail as follows:

Accuracy: Accuracy reflects the classifier’s ability to classify the whole sample, specifically, the overall evaluation metrics for determining positive samples as positive and negative samples as negative.

F1-score: The F1-score is known as the reconciled mean. Precision and recall have a negative correlation; when recall is bigger, precision is smaller. Therefore, we used the F1-score to reconcile precision and recall.

Precision: Precision is the proportion of true positive samples out of all samples predicted to be positive.

Recall: Recall is the proportion of all positive samples that are predicted to be positive. Theoretically, the value of recall is closer to 1, which indicates that the model is more predictive of performance.

### 6.3. Comparison Models

In order to validate the effectiveness of our model, we compared the CDAENet model with four state-of-the-art methods, including SVM [13], DT [14], CNN [15], and SCAE.

SVM [13]: This is the first and most used ML-based multipath signal recognition method. SVM is one of the most important traditional ML algorithms, with large advantages in prediction accuracy. The detection and removal of NLOS satellites using the methods in this article can achieve up to 87% classification accuracy.

DT [14]: This is one of the most classical ML methods and has also been used to attempt to detect MP and NLOS. In this article, we use the CART-type DT method.

CNN [15]: This is a DL method that uses CNN for feature extraction of data. The model can recognize both MP and NLOS in static and dynamic environments.

CSAE: Basic CAE was used to achieve data reduction and deep feature extraction, and LSTM was introduced into the model for learning the features of time series.

### 6.4. Training and Hyperparameter Tuning

**Figure 13:**
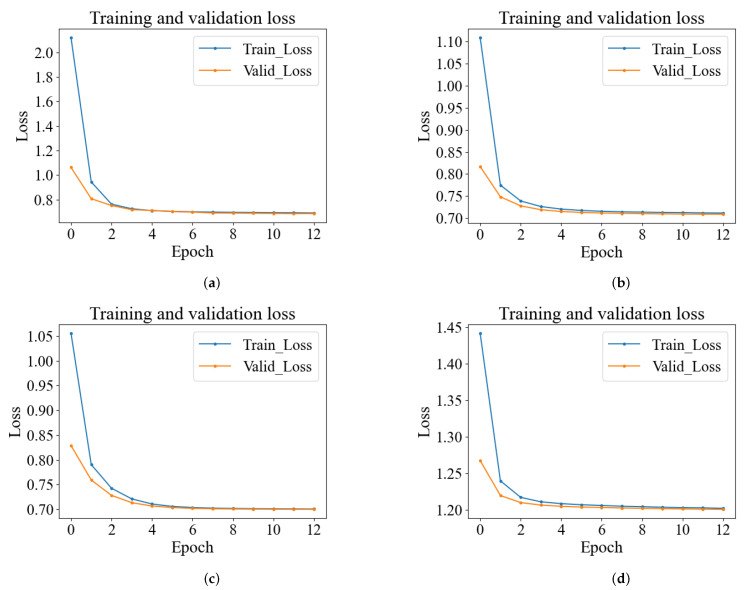
MSE loss function value progress during model training. (**a**) D1, (**b**) D2, (**c**) D3, (**d**) D4.

Usually, proper hyperparameter tuning of a DL model will help it achieve optimal detection performance. Therefore, we give the tuning procedure for the important parameter of the epoch. Figure 13 shows the test results on datasets D1, D2, D3, and D4. We give the loss function variation curves for the first 12 epochs of the training set versus the test set. At about 10 training times, the model converged, and the accuracy on the training and test sets is essentially maximal. As the training epoch increases, the loss basically stops decreasing. Therefore, we chose 10 as the appropriate epoch.

### 6.5. Comparison of Detection Performance

#### 6.5.1. Numerical Results Comparison

We have analyzed the performance of different detection models to demonstrate the effectiveness of this CDAENet model, as shown in Table 1, Table 2, Table 3 and Table 4. The following conclusions can be obtained from the experiment.

(a) Overall, the CDAENet detection model shows superior detection performance in terms of ACC, F1, Prec, and Rec compared to almost all baselines. For example, on the D1 dataset, CDAENet achieves 89.45%, 77.44%, 82.94%, and 70.36% for accuracy, F1-score, precision, and recall, respectively.
(b) It is worth noting that the value of recall is not high, which is mainly due to the fact that some NLOS samples are not detected. For urban forest environments where most of the samples are LOS, the CDAENet algorithm proposed in this article is still effective. Meanwhile, although the DT model performs well in all performance metrics, its detection accuracy is not as good as that of the proposed model. This may be because CDAENet classifiers can benefit from the deep feature extraction of BDS, which improves the detection performance.
(c) Compared with traditional ML models, CDAENet achieves very competitive and stable performance. Taking the dataset D1 as an example, CDAENet improves SVM and DT in detection accuracy by about 5.25% and 8.21%, respectively. The results show that the learned compression features of the BDS long-time series signals are effective. Speicifically, there is some information in the long-time BDS signals that is weakly helpful for the model to identify the signal type. On the contrary, the other part can be used as a key feature to identify its signal type, and our model effectively extracts this part of key features.
(d) The proposed CDAENet method consistently outperforms the DL-based methods CNN and CSAE, which demonstrates the effectiveness of learning time series correlation using DAE. Compared to the normal CNN model, the CDAENet method can extract more information between time series.
(e) The performance of DL-based methods is generally better than that of ML-based methods, which confirms that DL is more effective in learning data features.

#### 6.5.2. Visualization of the Detection Results

**Figure 14:**
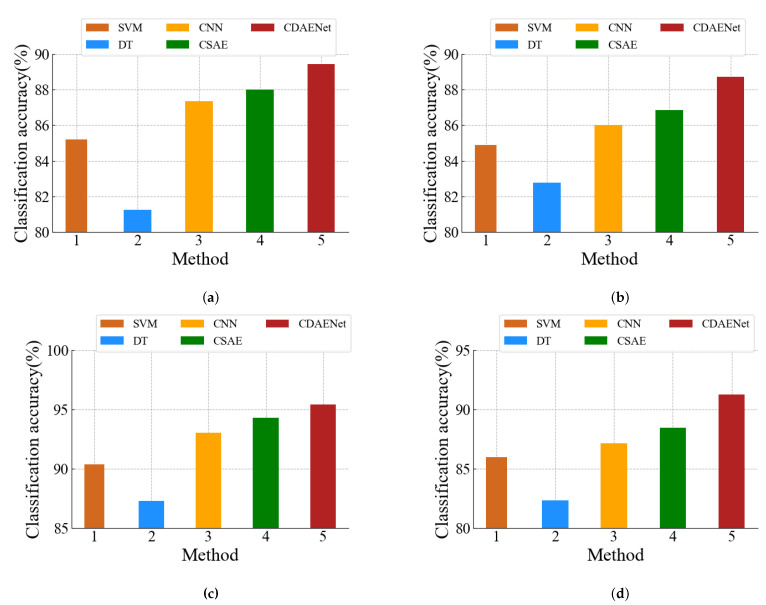
The BDS classification accuracy of different algorithms: (**a**) D1, (**b**) D2, (**c**) D3, (**d**) D4.

To assess the more intuitive overall performance of the CDAENet model, we give a visual analysis of the classification accuracy. Figure 14a–d show the results of the comparison of the D1–D4 data sets, respectively. The higher bars represent better overall detection performance of the model.

As shown in Figure 14, CDAENet has higher classification accuracy than the other four basic methods (i.e., SVM, DT, CNN, and SCAE) on all four datasets. Meanwhile, the classification performance of CNN and SCAE is better than that ofSVM and DT. This not only proves the superiority of our proposed model but also shows that the DL method is superior to the traditional ML methods.

### 6.6. The Sensitivity Analysis of the Noise

We added different noises, such as salt and pepper noise, uniform noise, and Gaussian noise, in our experiments. Table 5 gives the corresponding statistical results of the model classification accuracy on dataset D1 under different noises. As shown in Table 5, when salt and pepper noise, uniform noise, and Gaussian noise are added, the corresponding classification accuracies can reach 88.51%, 87.36%, and 89.45%, respectively. Among them, the classification accuracy obtained by the Gaussian noise method is higher than with the other methods, which indicates that the Gaussian noise method can effectively reduce the data noise and extract less redundant information.

In the case of adding noise, we analyze in detail the effect of the proposed CDAENet model, and the experimental results are shown in Table 6. Signal-to-noise ratios of 0 dB, 1 dB, 5 dB, 10 dB, 20 dB, 30 dB, and 40 dB Gaussian noise are added to the BeiDou satellite data to simulate the noise interference that accompanies the data in practical applications. The generalization and robustness of the features extracted by the DAE are slightly weaker when the noise signal has smaller values, such as 0 dB, 1 dB, and 5 dB, compared to the 10 dB noise, but the overall recognition rate is still maintained at a high level. As the noise signal increases further, it destroys the distribution of the original data to a certain extent, resulting in a decrease in the overall recognition rate. This shows that too much or too little noise can affect the performance of the DAE. Thus, adding a suitable noise signal can enhance the robustness of the model feature extraction.

### 6.7. Normalization Performance Evaluation

**Figure 15:**
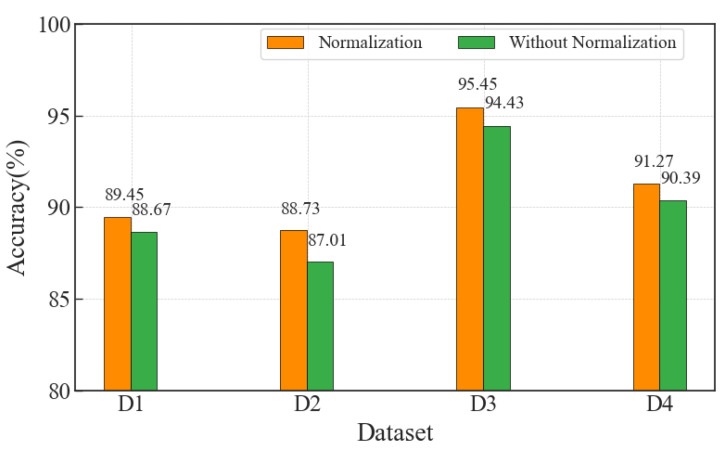
Normalization performance evaluation of D1, D2, D3 and D4.

To verify the effectiveness of normalization for our proposed CDAENet method, we plot quantitative experiments of normalization versus without normalization on the metric of classification accuracy. As shown in Figure 15, there is a significant improvement in the classification performance of the normalization method compared to without normalization. Specifically, the accuracy improved by 0.78%, 1.72%, 1.02%, and 0.88% for D1, D2, D3, and D4, respectively. This result proves that the normalization process is important in our method.

## 7. Discussion

In the experimental part, we validate the performance of our proposed CDAENet method under various sky-occlusion scenarios in an urban forest environment. The results show that our proposed CDAENet method not only converges quickly but also has good performance in all evaluation metrics. Compared with ML methods such as SVM and DT, we propose a more effective convolution-based DL method. In addition, unlike simple CNN and CSAE methods, the DAE approach effectively reduces the pollution of BDS data by environmental noise. Through the above analyses, we demonstrate the superiority of DAE in the recognition of NLOS in urban forest environments. DAE can effectively reduce the data dimension and weaken the noise of the data, so we can try to apply it to analyze the current hotspots of high-resolution remote sensing modern change detection [44] and the spatial–temporal distribution study of farmland [45]. In this article, we have analyzed and compared the proposed CDAENet method from various perspectives, such as convergence, classification performance, anti-interference ability, and normalization performance. Specifically, the detailed comparison between our proposed method and the existing methods is as follows.

(1) To compare the convergence performance of the proposed CDAENet method in detail, we provide the curves of epoch and loss under the MSE loss function for the training and validation sets. As shown in Figure 13, the convergence epochs of the proposed CDAENet method are basically the same on datasets D1, D2, D3, and D4 in the urban forest environment. When the epoch rises from 0 to 6, the loss decreases rapidly. At the same time, when the epoch rises from 6 to 10, loss essentially stops decreasing. To further verify this trend, we trained 12 epochs, at which point loss remained essentially unchanged relative to 10. Therefore, we can infer that 10 is the best epoch. At this point, the model shows excellent performance on all four datasets.
(2) To demonstrate the superior classification performance of the CDAENet method, we conducted comparison experiments with the ML methods SVM and DT on four datasets. The comparison results are shown in Table 1, Table 2, Table 3 and Table 4. On the four datasets, the CDAENet method outperformed the baseline learning method on most of the ACC, F1, Prec, and Rec metrics. Among them, the best performance is on dataset D3, which outperformed all baseline methods with ACC, F1, Prec, and Rec of 95.45%, 74.33%, 86.56%, and 73.21%, respectively. Meanwhile, we also observed that existing DL methods, such as the CNN and CSAE methods, outperform ML methods such as SVM and DT. Therefore, DL methods are more feasible than ML methods in solving the NLOS recognition problem. It is worth noting that the individual F1-score and recall values are not high, which is mainly due to the fact that some NLOS samples are not detected. For urban forest environments where most of the samples are LOS, the CDAENet method proposed in this paper is still valid. Compared to other metrics, classification accuracy is more important for this study. As shown in Figure 14, our proposed model outperforms other methods in classification accuracy across all metrics.
(3) We report the performances of the CDAENet method for various noises with the best corresponding parameters to present their robustness against noises. As shown in Table 5, when we added salt and pepper noise, uniform noise, and Gaussian noise to the model, the classification accuracies on dataset D1 were 88.51%, 87.36%, and 89.45%, respectively. Therefore, we chose to add Gaussian noise to the model. As shown in Table 6, adding Gaussian noise to the original BDS signal at 0dB, 1dB, 5dB, 10dB, 20dB, 30dB, and 40dB corresponds to classification accuracies of 86.51%, 87.37%, 88.96%, 89.45%, 88.75%, 88.21%, and 87.41%, respectively. From the results, it can be seen that the CDAENet method performs better under 10 dB noise, and the classification accuracy can reach 89.45%. This is because the CDAENet method can effectively learn the features containing noisy data during the training process and improve its anti-interference ability. However, the performance is poorer when the BDS signal is almost completely flooded by noise, i.e., 20 dB, 30 dB, and 40 dB noise. Therefore, it can be concluded that filling in the appropriate Gaussian noise can effectively improve the classification performance of the model.
(4) The normalization performances of different datasets are analyzed in Figure 15. The proposed normalization can always obtain the best classification performances in different noise conditions and different environments. In detail, normalization improves over no normalization by at least 0.78% and at most tby 1.72% on the four datasets, indicating that normalization is particularly important for our proposed model.

## 8. Conclusions

In this article, we propose an unsupervised CDAENet method, which can effectively extract time series information based on DAE, to solve the problem of low recognition accuracy for BeiDou satellite NLOS signal in urban forest environments. The proposed method iteratively trains the model by minimizing the mean absolute error loss. DAE can achieve high-dimensional data compression while preserving potential key features of time series data. To improve the robustness of NLOS signal detection in BDS, we added Gaussian noise to the autoencoder to reduce the sensitivity of the model to the input data. Furthermore, to improve the detection performance of the model, we used the MLP method to detect the compressed nonlinear data. Therefore, we constructed the DAE-based DL model CDAENet to improve the classification performance of NLOS signals in urban forest environments.

We collected noise-containing BDS signals from urban forest environments under four different sky-occlusion scenarios and validated the superiority of the CDAENet model through a variety of evaluation metrics. The experimental results show that the CDAENet-based method proposed in this article can converge quickly in all four urban forest environments. Our method essentially stops changing after 10 epochs. This result indicates that our model has a relatively fast convergence. Meanwhile, our proposed method has better classification results. Specifically, the classification accuracy is improved by 5.25% and 8.21% when compared to two well-known ML algorithms, SVM and DT, respectively. In particular, our proposed CDAENet method can achieve more than 95% classification accuracy and generally outperforms the benchmark methods in terms of F1-score, precision, and recall. In addition, the classification accuracy is improved by 8% and 3% over DL-based and ML-based methods, respectively. Notably, it solves the complex problem of classifying noise-laden BDS signals in practical engineering. Regarding the addition of different noise components, we tried the types of salt and pepper noise, uniform noise, and Gaussian noise. The statistical performance shows that Gaussian noise is the most effective. Filling in the appropriate noise can effectively improve the classification accuracy of the model, and adding 10 dB of noise improves the classification accuracy by about 2.94% and 2.04% compared with no noise and adding 40 dB of noise, respectively. The results show that the method effectively reduces the pollution of environmental noise during the training process and can further improve the classification performance under various sky-obscured environments in urban forests. In addition, urban forests have less signal masking, and environmental noise is the main reason affecting signal recognition. DAE can effectively mitigate environmental noise interference. Comparatively, mountainous areas or urban canyons are highly obstructed, and noise is not the most important influencing factor. Therefore, DAE can also be used in mountainous areas and urban canyons, but there may be more suitable methods to solve the NLOS recognition problem in these cases. Meanwhile, the normalization method can achieve higher classification accuracy than without normalization on the four datasets, and this result proves the importance of normalization.

In our future work, we will continue to study deep-learning-based multipath signal recognition in complex urban environments. We are committed to applying it to richer complex urban environments (e.g., urban canyons, urban overpasses, andd mountains). In addition, we have noticed that CDAENet has good anti-interference ability for BDS signal extraction, but the recognition efficiency and generalization performance of the model still need to be improved. To overcome this limitation, we plan to investigate more types of AEs for better extraction of key features, such as the variational autoencoder and the graph autoencoder.

## Figures and Tables

**Table 1:** Performance comparison between CDAENet and other methods on the D1 dataset.

Method	Evaluation Index			
	**Accuracy (%)**	**F1-Score (%)**	**Precision (%)**	**Recall (%)**
SVM [13]	85.20	46.94	70.13	50.41
DT [14]	81.24	75.19	74.77	75.64
CNN [15]	87.35	61.58	75.41	58.87
CSAE	88.81	67.96	79.48	63.48
**CDAENet**	**89.45**	**77.44**	**82.94**	70.36

The number in bold denotes it is the best result.

**Table 2:** Performance comparison between CDAENet and other methods on the D2 dataset.

Method	Evaluation Index			
	**Accuracy (%)**	**F1-Score (%)**	**Precision (%)**	**Recall (%)**
SVM [13]	84.89	47.06	44.44	50.00
DT [14]	82.78	57.62	57.58	56.66
CNN [15]	86.01	50.48	74.40	51.65
CSAE	86.87	53.10	77.64	52.99
**CDAENet**	**88.73**	55.53	**78.06**	54.09

The number in bold denotes it is the best result.

**Table 3:** Performance comparison between CDAENet and other methods on the D3 dataset.

Method	Evaluation Index			
	**Accuracy (%)**	**F1-Score (%)**	**Precision (%)**	**Recall (%)**
SVM [13]	90.38	57.91	72.48	55.42
DT [14]	87.29	71.27	71.63	71.48
CNN [15]	93.06	63.46	85.36	61.70
CSAE	94.29	67.47	86.40	65.47
**CDAENet**	**95.45**	**74.33**	**86.56**	**73.21**

The number in bold denotes it is the best result.

**Table 4:** Performance comparison between CDAENet and other methods on the D4 dataset.

Method	Evaluation Index			
	**Accuracy (%)**	**F1-Score (%)**	**Precision (%)**	**Recall (%)**
SVM [13]	85.99	47.64	45.50	50.00
DT [14]	82.30	68.14	67.72	68.60
CNN [15]	87.13	58.17	72.25	55.99
CSAE	88.45	63.44	72.94	59.36
**CDAENet**	**91.27**	65.01	**74.65**	63.31

The number in bold denotes it is the best result.

**Table 5:** Model under different types of noise components.

Noise	Accuracy
Salt and Pepper Noise	88.51%
Uniform Noise	87.36%
Gaussian Noise	**89.45%**

The number in bold denotes it is the best result.

**Table 6:** Model under different noise components.

**Noise**	0 dB	1 dB	5 dB	10 dB	20 dB	30 dB	40 dB
**Accuracy**	86.51%	87.37%	88.96%	**89.45**%	88.75%	88.21%	87.41%

The number in bold denotes it is the best result.

## Data Availability

Data are contained within the article.
